# The Impact of Social Determinants of Health on Discharge Disposition Following One- and Two-Level Posterior Interbody Fusion

**DOI:** 10.7759/cureus.52939

**Published:** 2024-01-25

**Authors:** Michelle A Zabat, Lindsay Kim, Priscilla P Varghese, Brooke K O'Connell, Yong H Kim, Charla R Fischer

**Affiliations:** 1 Orthopaedic Surgery, New York University (NYU) Grossman School of Medicine, New York, USA; 2 Orthopaedic Surgery, State University of New York (SUNY) Downstate Health Sciences University, College of Medicine, Brooklyn, USA

**Keywords:** posterior lumbar interbody fusion (plif), transforaminal lumbar interbody fusion (tlif), discharge disposition, socioeconomic disparities, social determinants of health (sdoh)

## Abstract

Background

Current research is limited in exploring the impact of social determinants of health (SDOH) on the discharge location within elective spine surgery. Further understanding of the influence of SDOH on disposition is necessary to improve outcomes. This study explores how SDOH influence discharge disposition for patients undergoing one- or two-level posterior interbody fusion (TLIF/PLIF).

Methods

This was a retrospective propensity-matched cohort study*. *Patients who underwent TLIF/PLIF between 2017 and 2020 at a single academic medical center were identified. The chart review gathered demographics, perioperative characteristics, intra/post-operative complications, discharge disposition, and 90-day outcomes. Discharge dispositions included subacute nursing facility (SNF), home with self-care (HSC), home with health services (HHS), and acute rehab facility (ARF). Demographic, perioperative, and disposition outcomes were analyzed by chi-square analysis and one-way ANOVA based on gender, race, and income quartiles.

Results

Propensity score matching for significant demographic factors isolated 326 patients. The rate of discharge to SNF was higher in females compared to males (25.00% vs 10.56%; p=0.001). Men were discharged to home at a higher rate than women (75.4% vs 61.95%; p=0.010). LatinX patients had the highest rate of home discharge, followed by Asians, Caucasians, and African Americans (83.33% vs 70.31% vs 66.45% vs 65.90%; p<0.001). The post hoc Tukey test demonstrated statistically significant differences between Asians and all other races in the context of age and BMI. Additionally, patients discharged to SNF showed the highest Charlson comorbidity index (CCI) score, followed by those at ARF, HHS, and HSC (4.36 vs 4.05 vs 2.87 vs 2.37; p<0.001). The estimated median income for the cohort ranged from $52,000 to $250,001, with no significant differences in income seen across comparisons.

Conclusion

Discharge disposition following one- or two-level TLIF/PLIF shows significant association with gender and race. No association was seen when comparing discharge rates among zip code-level median income quartiles.

## Introduction

Transforaminal lumbar interbody fusion and posterior lumbar interbody fusion (TLIF/PLIF) are widely established surgical options indicated for spinal degenerative and structural pathologies such as spondylolisthesis, scoliosis, spinal stenosis, fractures, infections, and herniations [1]. Considering the aging population and advances in surgical techniques, the demand for these surgeries is expected to continue rising, with volumes of elective lumbar fusion already increasing to 62.3% between 2004 and 2015 [2-4]. Therefore, it is imperative to investigate understudied risk factors associated with adverse TLIF/PLIF surgical outcomes, particularly discharge disposition, which is known to be influenced by social determinants of health (SDOH).

Discharge disposition often serves as an indicator of post-surgical functional outcomes and is a major contributor to the total cost of care. Specifically, studies have shown that disposition to non-home locations following lumbar fusion surgery is associated with post-operative complications, including higher rates of mortality, readmission, length of stay, unplanned reoperations, and post-discharge complications such as sepsis-related complications, wound complications, and deep venous thrombosis [5-9]. Risk factors for non-home discharge disposition include increased age, increased body mass index (BMI), female sex, more than 10% weight loss six months prior to surgery, and a history of comorbidities such as chronic obstructive pulmonary disease or diabetes [5-6,9-10]. Few studies, however, explore the relationship between discharge location and socioeconomic status (SES) status within the context of elective spine surgery, specifically TLIFs and PLIFs, highlighting a clear paucity in the available orthopedic literature [8,10].

SES is a complex social force that comprises an individual’s perceived social class, social status, and quality of life, among other influences [11]. These components are underlain by SDOH, defined by the WHO as the conditions in which people are born, grow, live, work, and age [12]. These are critically important considerations in orthopedics, as SES and SDOH are proven to predict morbidity, mortality, and care quality [13]. In the present study, we evaluate the impact of sex, self-reported race, and SES, all of which are proven important SDOH.

Given that the existence of health inequities within healthcare has been well established and has frequently been associated with unfavorable outcomes for minority patients, exploring SES status as a potential contributor to dismissal to specific non-home locations such as skilled facility or acute rehabilitation following lumbar fusion unit warrants further investigation. Therefore, the purpose of this study was to primarily identify the implications of SES disparities on discharge dispositions in the setting of one- or two-level TLIF/PLIF. Secondary outcomes explored include rates associated with emergency department visits and readmissions. We hypothesize that SES and demographic factors will affect the discharge disposition of patients who undergo TLIF/PLIF.

## Materials and methods

Study design

Patients aged 18 or older who underwent primary elective one- or two-level TLIF or PLIF between 2017 and 2020 were identified from a single academic medical center. Subjects were excluded for urgent or emergent procedures, revision surgery, or if they had worker’s compensation, no-fault insurance, or a race of “Unknown” or “Other” listed in their medical records. Patients whose mailing addresses were outside the U.S. or did not correspond to a zip code reporting income to the Census Bureau were excluded as their approximated income could not be readily assessed.

Data collection

A chart review was performed for demographics, body mass index (BMI), smoking status, intra-operative complications, post-operative complications, discharge disposition, readmissions, and post-operative emergency department (ED) visits. Discharge dispositions included subacute nursing facility (SNF), home with self-care (HSC), home with health services (HHS), and acute rehab facility (ARF). Disposition was additionally categorized more broadly as home (HSC/HHS) vs. non-home (SNF/ARF). 

Median income values were approximated by five-digit patient home zip codes using data from the U.S. Census Bureau and American Community Survey 2018. Given the complexity of measuring SES, in practice, SES is often represented as a composite measure accounting for influences such as educational achievement, income, or occupation [14]. Income, especially for retrospective studies, is often captured by using median income for an individual’s home zip code as a proxy measure, though care should be taken to account for the limitations of this approach [15].

Statistical analysis

SDOH of interest were defined as self-reported race, gender, and SES based on known relationship to patient outcomes. Propensity score matching was performed according to demographics which differed significantly at baseline between the analysis cohorts. More specifically, analysis based on sex was matched for age and BMI; analysis of race was matched for age, sex, BMI, and smoking status; and the analysis of income was matched for sex. Primary endpoints of this study were to analyze relationships between SES factors, demographics, discharge disposition, 90-day medical complications, 90-day readmission rates, and 90-day emergency room visits. 90-day medical complications included: cardiac complications, neurological complications, pulmonary complications, deep vein thromboses/pulmonary embolism, ileus, urinary complications, deep surgical site infections, and mechanical complications.

Statistical analyses were performed using IBM SPSS Statistics for Windows, Version 24 (Released 2016; IBM Corp., Armonk, New York, United States). Chi-square analyses and one-way ANOVA were used to compare baseline demographics including age, gender, BMI, and smoking status for the cohorts in each of the three analyses. The cohorts were assessed in the context of approximated median income, race, and gender, and by chi-square, one-way ANOVA, and t-test where appropriate. Relationships between SES factors, demographics, discharge dispositions, 90-day complications, 90-day readmissions, and 90-day ED visits were analyzed via chi-square analyses and one-way ANOVA. Post-hoc analysis was performed using the Tukey test to identify specific differences between analysis cohorts. Significance was set to p<0.05.

## Results

Demographic analysis

Initial query identified 635 patients who underwent one- or two-level TLIF/PLIF surgeries. Propensity score matching further isolated 326 patients who had undergone either one-level (n=235) or two-level (n=91) TLIF/PLIF procedures; 56.4% (n=184) of the cohort was female and 41.1% (n=134) had a positive smoking history. The average age was 64.26 years, average BMI was 28.95 kg/m^2^, and the median income based on zip code-level data was $82,045.62. Quartile ranges for medial income values based on zip code-level data were defined as $52,000-57,010 (28.4%, n=93); $57,011-80,893 (26.6%, n=87); $80,894-108,952 (29.9%, n=97); $108,953-250,001 (25.0%, n=82). The cohort racial demographics were as follows: 54.9% Caucasian (n=179); 13.2% African American (n=43); 18.1% Asian (n=59); and 8.9% LatinX (n=29) (Table 1). Surgical variables included an average LOS of 3.9 days, ASA of 2.5, and estimated blood loss of EBL of 340.66mL.

**Table 1:** Patient Demographics After Propensity Score Matching Continuous data is represented by means. Categorical data is represented by % (N). BMI: Body mass index; TLIF/PLIF: one- or two-level posterior interbody fusion.

One- and Two-Level TLIF/PLIF	n= 326
Average Age (years)	64.26
Average BMI (kg/m^2^)	28.95
Average Approximate Median Income ($)	82,045.62
Positive Smoking hx. (%)	41.1% (134)
Female (%)	56.4% (184)
Caucasian (%)	54.9% (179)
African American (%)	13.2% (43)
LatinX (%)	8.9% (29)
Asian (%)	18.1% (59)

Sex analysis

Female patients were older and had significantly greater BMI in comparison to male patients (Age: 64.17 vs 63.7 years; p=0.03; BMI: 29.03 vs 28.84 kg/m^2^; p<0.001). Female patients also had higher rates of cardiac complications (10.9% (n=2) vs 2.8% (n=4); p=0.005), lower rates of urinary complications (2.7% (n=5) vs 8.59% (n=12); p=0.025), and lower rates of neurological complications (2.7% (n=5) vs 8.5% (n=12); p=0.025) following TLIF/PLIF. The rate of discharge to SNF was higher in female patients compared to male patients (25.0% (n=46) vs 10.6% (n=15); p=0.001) following TLIF/PLIF. No significant differences were seen in rates of discharge to HSC, HHS, or ARF. When comparing home vs non-home disposition, female patients were found to be discharged to home at a lower rate than male patients (61.95% (n=114) vs 75.4% (n=107) vs; p=0.010). When evaluating for differences in ED visits, female patients showed lower rates than male patients (1.1% (n=2) vs 7.0% (n=10); p=0.006). No significant differences were seen in rates of readmission between the sexes (Table 2).

**Table 2:** Comparison of Sex by Demographics, Complications, and Discharge Disposition Continuous data is represented by means. Categorical data is represented by % (N). Statistical significance is set at p<0.05; significant results are in bold. BMI: Body mass index; EBL: estimated blood loss; ASA: American Society of Anesthesiologists score; DVT/PE: deep vein thrombosis / pulmonary embolism; SSI: surgical site infection; OR: operating room; ED: emergency department.

		Differences between Sexes		
		Male N=142	Female N=184	p-value
Demographics	Age (years)	63.7	64.17	0.03
	Sex	N/A	N/A	N/A
	BMI (kg/m^2^)	28.84	29.03	<0.001
	Smoker (%)	73.4% (104)	67.0% (123)	0.344
	Procedure and Admission			
	Length of Stay (days)	3.64	4.13	0.085
	Levels Fused	1.26	1.29	0.513
	EBL (mL)	340.54	340.75	0.995
	ASA	2.43	2.51	0.218
	Incidence of Intra-op Complication (%)	8.4% (12)	14.6% (27)	0.371
	Incidence of Durotomy (%)	2.8% (4)	6.0% (11)	0.196
	Incidence of Significant Blood Loss (%)	0.7% (1)	0.0% (0)	0.4356
	Incidence of Post-op Complication	N/A	N/A	N/A
	Incidence of Cardiac Complication (%)	2.8% (4)	10.9% (2)	0.005
	Incidence of Neurologic Complication (%)	8.5% (12)	2.7% (5)	0.025
	Incidence of Pulmonary Complication (%)	2.1% (3)	3.3% (6)	0.737
	Incidence of DVT/PE (%)	0.7% (1)	0.0%	0.436
	Incidence of Ileus (%)	4.2% (6)	2.2% (4)	0.341
	Incidence of Urinary Complication (%)	8.5% (12)	2.7% (5)	0.025
	Incidence of Deep SSI (%)	1.4% (2)	3.3% (6)	0.526
	Incidence of Mechanical Complication (%)	0.7% (1)	2.2% (4)	0.509
	Return to OR in 30 Days (%)	1.4% (2)	2.7% (5)	0.703
	Return to OR in 90 days (%)	2.1% (3)	4.9% (9)	0.242
Post-Discharge	Discharge Disposition			
	Skilled Nursing Facility (%)	10.6% (15)	25.0% (46)	0.001
	Home - under care of Home Health Services (%)	59.2% (84)	52.2% (96)	0.219
	Home or Self-care (%)	16.2% (23)	9.8% (18)	0.083
	Acute Rehab Facility (%)	7.7% (11)	5.4% (10)	0.496
	Home (%)	75.0% (107)	62.0% (114)	0.010
	Non-Home (%)	25.0% (35)	38.0% (70)	0.010
	Readmission (%)	2.1% (3)	4.3% (8)	0.358
	ED visit (%)	7.0% (10)	1.1% (2)	0.006

Self-reported race analysis

Asian patients were found to have the highest CCI score, followed by African American patients, LatinX patients, and Caucasian patients (3.76 vs 3.06 vs 2.88 vs 2.84; p=0.008). Incidence of ileus was highest in LatinX patients (p=0.042). The post hoc Tukey test was performed and demonstrated statistically significant differences between Asian patients and all other races for age and BMI. Asian patients and African American patients showed the greatest mean differences for age (8.41 years; p<0.01) and BMI (6.13 kg/m^2^; p<0.001). No significant differences in discharge disposition to SNF, HSC, HHS, or ARF were found. LatinX patients had the highest rate of home discharge, followed by Asian patients, Caucasian patients, and African American patients (83.33% (n=24) vs 70.31% (n=41) vs 66.45% (n=119) vs 65.90% (n=28); p<0.001). Additionally, no significant difference in rates of readmission or ED visits were found. The post hoc Tukey test was not performed given the lack of significant findings on ANOVA (Table 3).

**Table 3:** Comparison of Self-Reported Race by Demographics, Complications, and Discharge Disposition Continuous data is represented by means. Categorical data is represented by % (N). Statistical significance set a p<0.05; significant results are in bold. BMI: Body mass index; EBL: estimated blood loss; ASA: American Society of Anesthesiologists score; DVT/PE: deep vein thrombosis/pulmonary embolism; SSI: surgical site infection; OR: operating room; ED: emergency department.

		Differences between Self-reported Races				
		Caucasian N=179	African American N=43	LatinX N=29	Asian N=59	p-value
Demographics	Age (years)	64.46	61.42	61.790	69.83	<0.001
	Sex (% female)	51.4% (92)	44.2% (19)	53.7% (16)	59.3% (35)	<0.001
	BMI (kg/m^2^)	29.27	31.48	30.36	25.38	<0.001
	Smoker	45.8% (82)	76.7% (33)	58.6% (17)	71.2% (42)	<0.001
	Procedure and Admission					
	Length of Stay (days)	4.03	4.35	3.6	3.47	0.300
	Levels Fused	1.3	1.23	1.28	1.31	0.828
	EBL (mL)	372.95	324.05	268.97	318.98	0.190
	ASA	2.52	2.44	2.52	2.39	0.523
	Incidence of Intra-op Complication (%)	9.5% (17)	4.7% (2)	3.4% (1)	1.7% (1)	0.149
	Incidence of Durotomy (%)	6.7% (12)	4.7% (2)	0.0% (0)	1.7% (1)	0.251
	Incidence of Significant Blood Loss (%)	0.6% (1)	0.0% (0)	0.0% (0)	0.0% (0)	0.865
	Incidence of Post-op Complication (%)	29.6% (53)	20.9% (9)	27.6% (8)	18.6% (11)	0.325
	Incidence of Cardiac Complication (%)	8.4% (15)	4.7% (2)	10.3% (3)	5.1% (3)	0.671
	Incidence of Neurologic Complication (%)	6.1% (11)	7.0% (3)	0.0% (0)	3.5% (2)	0.457
	Incidence of Pulmonary Complication (%)	3.4% (6)	0.0% (0)	3.4% (1)	3.5% (2)	0.684
	Incidence of DVT/PE (%)	0.6% (1)	0.0% (0)	0.0% (0)	0.0% (0)	0.865
	Incidence of Ileus (%)	2.2% (4)	4.7% (2)	10.3% (3)	0.0% (0)	0.042
	Incidence of Urinary Complication (%)	5.6% (10)	7.0% (3)	6.9% (2)	3.5% (2)	0.85
	Incidence of Deep SSI (%)	2.8% (5)	2.3% (1)	0.0% (0)	0.0% (0)	0.488
	Incidence of Mechanical Complication (%)	3.4% (6)	0.0% (0)	0.0% (0)	0.0% (0)	0.612
	Return to OR in 30 Days (%)	3.4% (6)	0.0% (0)	0.0% (0)	0.0% (0)	0.214
	Return to OR in 90 days (%)	5.6% (10)	2.3% (1)	3.4% (1)	0.0% (0)	0.253
Post-Discharge	Discharge Disposition					
	Skilled Nursing Facility (%)	16.2% (29)	23.3% (10)	13.8% (4)	30.5% (18)	0.081
	Home - under care of Home Health Services (%)	57.5% (103)	53.5% (23)	75.9% (22)	54.2% (32)	0.211
	Home or Self-care (%)	14.0% (25)	14.0% (6)	6.9% (2)	13.6% (8)	0.771
	Acute Rehab Facility (%)	8.9% (16)	2.3% (1)	3.4% (1)	1.7% (1)	0.236
	Home (%)	66.4% (119)	65.9% (28)	83.3% (24)	70.3% (41)	<0.001
	Non-Home (%)	33.6% (60)	34.1% (15)	16.7% (5)	29.7% (18)	<0.001
	Readmission (%)	5.6% (10)	2.3% (1)	0.0% (0)	0.0% (0)	0.133
	ED visit (%)	5.0% (9)	2.3% (1)	6.9% (2)	0.0% (0)	0.260

Estimated income class quartile analysis

No significant differences in demographic factors were seen between zip code data-based income class quartiles. No significant differences in discharge disposition to SNF, HSC, HHS, or ARF were noted. Differences were also not found for HSC/HHS vs. SNF/ARF disposition. Rates of readmission or ED visits among quartiles were also found to show no statistical difference. The post hoc Tukey test was not performed for this analysis due to the lack of significant findings on ANOVA (Table 4).

**Table 4:** Quartiles of Median Income by Demographics, Complications, and Discharge Disposition Continuous data is represented by means. Categorical data is represented by % (N). Statistical significance set at p<0.05; significant results are in bold. BMI: Body mass index; EBL: estimated blood loss; ASA: American Society of Anesthesiologists score; DVT/PE: deep vein thrombosis/pulmonary embolism; SSI: surgical site infection; OR: operating room; ED: emergency department.

		Differences Between Quartiles of Median Income for Cohort				
		1st N=91	2nd N=82	3rd N=67	4th N=80	p-value
Demographics	Age (years)	65.1	64.1	64.93	62.86	0.623
	Sex (% female)	68.0% (62)	51.2% (42)	46.3% (31)	56.3% (45)	0.029
	BMI (kg/m^2^)	28.84	29.34	29.74	28.01	0.349
	Smoker (%)	62.0% (56)	63.3% (52)	65.9% (44)	77.1% (62)	0.124
	Procedure and Admission					
	Length of Stay (days)	3.84	4.36	4	3.51	0.193
	Levels Fused	1.29	1.23	1.34	1.26	0.489
	EBL (mL)	345.11	308.27	334.69	373.48	0.505
	ASA	2.48	2.5	2.42	2.45	0.85
	Incidence of Intra-op Complication (%)	3.1% (3)	3.7% (3)	10.4% (7)	10.0% (8)	0.097
	Incidence of Durotomy (%)	3.1% (3)	2.4% (2)	9.0% (6)	5.0% (4)	0.231
	Incidence of Significant Blood Loss (%)	0.0% (0)	0.0% (0)	0.0% (0)	1.3% (1)	0.379
	Incidence of Post-op Complication (%)	22.7% (21)	32.9% (27)	28.4% (19)	22.5% (18)	0.355
	Incidence of Cardiac Complication (%)	6.2% (6)	7.3% (6)	9.0% (6)	7.5% (6)	0.93
	Incidence of Neurologic Complication (%)	7.2% (7)	3.7% (3)	7.5% (5)	2.5% (2)	0.382
	Incidence of Pulmonary Complication (%)	1.0% (1)	6.1% (5)	3.0% (2)	1.3% (1)	0.159
	Incidence of DVT/PE (%)	0.0% (0)	0.0% (0)	0.0% (0)	1.3% (1)	0.379
	Incidence of Ileus (%)	3.1% (3)	3.7% (3)	4.5% (3)	1.3% (1)	0.698
	Incidence of Urinary Complication (%)	6.2% (6)	4.9% (4)	3.0% (2)	6.3% (5)	0.789
	Incidence of Deep SSI (%)	1.0% (1)	4.9% (4)	3.0% (2)	1.3% (1)	0.201
	Incidence of Mechanical Complication (%)	1.0% (1)	1.2% (1)	3.0% (2)	2.5% (2)	0.876
	Return to OR in 30 Days (%)	1.0% (1)	3.7% (3)	1.5% (1)	2.5% (2)	0.648
	Return to OR in 90 days (%)	3.1% (3)	4.9% (4)	1.5% (1)	5.0% (4)	0.632
Post-Discharge	Discharge Disposition					
	Skilled Nursing Facility (%)	25.8% (23)	20.7% (17)	14.9% (10)	11.3% (9)	0.073
	Home - under care of Home Health Services (%)	52.6% (48)	48.8% (40)	52.2% (35)	67.5% (54)	0.080
	Home or Self-care (%)	8.2% (8)	18.3% (15)	16.4% (11)	8.8% (7)	0.109
	Acute Rehab Facility (%)	7.8% (7)	7.3% (6)	7.5% (5)	3.8% (3)	0.734
	Home (%)	65.0% (59)	69.0% (57)	74.0% (50)	79.0% (63)	0.193
	Non-Home (%)	35.0% (32)	31.0% (25)	26.0% (17)	21.0% (17)	0.193
	Readmission (%)	3.1% (3)	4.9% (4)	1.5% (1)	3.8% (3)	0.716
	ED visit (%)	5.2% (5)	4.9% (4)	0.0% (0)	3.8% (3)	0.322

## Discussion

Discharge disposition is an important component of post-operative outcomes following spine surgery. Numerous adverse surgical outcomes have been associated with non-home discharges, such as higher mortality and readmission rates and greater lengths of stay, thus an increase in hospital costs [8,9]. Given the potential impact of discharge disposition on a patient’s quality of life, it is important to understand the factors that influence it, which include SDOH and SES. Though prior studies have evaluated the impact of SES on disposition, many limit their analysis of disposition locations to home versus non-home, without accounting for the variety of non-home locations (SNF vs. ARF) or differentiating between HSC or HHS [8-10]. This present study seeks to fill this gap by assessing the impact of SDOH including SES, sex, and self-reported race on specific discharge disposition.

Chiefly, we found that discharge disposition following TLIF/PLIF exhibits a significant relationship with gender and race but not with zip code-level income class quartile. Sex disparities have been shown to influence post-operative outcomes with female patients reported to have increased length of hospital stay, lower long-term satisfaction scores, and increased hospital spending due to complications following spine surgery [13,16-21]. Prior work assessing the influence of sex on disposition has been inconclusive, with some studies suggesting increased non-home disposition for women and others finding no difference [18,22,23]. The present study adds to the body of literature suggesting female patients are discharged to home at a lower rate than men (61.95% (n=114) vs 75.4% (n=107); p=0.010). However, similar to the self-reported race-stratified cohorts, the female cohort in this study had a greater average age and average BMI compared to the male cohort. Given these discrepancies, it is important to consider these confounding influences [8-10,22-26].

Compared to other studies evaluating spine surgery patients, our cohort demographics trended toward greater diversity in self-reported race. Our cohort showed a breakdown of 54.9% Caucasian (n=179); 13.2% African American (n=43); 18.1% Asian (n=59); and 8.9% LatinX (n=29); comparable studies show a proportion of Caucasian patients around 70 - 80% [27]. This likely reflects the location of the study institution in New York City, which is reported to have a population estimated at 42.7% White according to July 2021 United States Census Bureau data. Self-reported race has also previously been identified as a risk factor predictive of poor outcomes and discharge disposition after lumbar spine surgery [28]. Numerous studies support that Black patients tend to see higher rates of non-home health facility discharge following spine surgery [10,24]. Our study results echo these findings, with Black patients exhibiting the highest rate of non-home discharge, followed by Caucasian, Asian, and LatinX patients. However, we found no significant differences when disposition was further stratified into SNF, HSC, HHS, or ARF. This is surprising considering the well-established associations between self-reported race and discharge disposition, as well as the fact that significant differences have been found for similar comparisons after total knee arthroplasty or total hip arthroplasty [29]. However, it is important to note that the cohorts in this study had significant differences in age, sex, BMI, and smoking status, as these factors are known to be independent predictors of non-home discharge. Given the possibility that these variables may impact discharge disposition, further analysis by self-reported race after controlling for these influences would be valuable and should be pursued in future studies [8-10,22-26].

The lack of significant findings in the income analysis was surprising given the well-established association between SES and discharge disposition [8]. A possible explanation for the discordant results of the present study is the influence of New York City’s economic environment. For example, the current study population had an average median zip code-level income of $82,045.62; this is relatively similar to the median zip code-level income of $67,997 reported for New York City as a whole in 2021 [30]. Our quartiles ranges were $52,000 - $57,010 (28.4%, n=93), $57,011 - $80,893 (26.6%, n=87), $80,894 - $108,952 (29.9%, n=97), and $108,953 - $250,001 (25.0%, n=82). The meaningfully wealthier population captured in our study may arise from a variety of influences. It may be a reflection of not only the population that inhabits New York City but also the proportion of this population that is cared for at the study institution, a tertiary care center in the Manhattan borough. Given this context, an important consideration is that this analysis excludes patients undergoing TLIF/PLIF who come from a more impoverished socioeconomic class. This may contribute to the lack of differences seen in discharge disposition. Further similar studies at different institutions in New York City, as well as in geographies outside of New York City, are necessary to better understand these economic influences.

The present study is not without limitations. In addition to the limitations described previously regarding the idiosyncrasies of New York City’s economic environment, there are also limitations associated with SES evaluation related to the use of zip code-level median income data. Though this method of assessing SES is widely used and accepted, inherent caution should be used when extrapolating neighborhood-level data to make conclusions on an individual patient level, given that it cannot accurately represent every individual's financial situation. Additionally, it is important to note that the current study was conducted at a large single academic medical center in the Northeast region of the United States. Northeastern geography and a larger number of beds associated with the treating hospital have been shown to be independent predictors of non-home discharge, and so these confounding influences should be taken into consideration as well when extrapolating our results [8]. Racial representation can also be considered a limitation since the majority of the patients in our study cohort were Caucasian (54%, n=179), followed by Asian (18.1%, n=59), African American (13.2%, n=43), and LatinX (8.9%, n=29). Though this demographic breakdown is more diverse with regard to minority identity when compared to the majority of other studies assessing discharge disposition after spinal surgery, this still reflects limited heterogeneity with regard to minority races; the smaller sample sizes in the minority demographic groups may limit the power of comparisons made for these cohorts. Also, while sex, self-reported race, and SES are well-established as meaningful SDOH, there are other SDOH not evaluated in this study (e.g., educational attainment, health insurance status) which may serve as confounders. Finally, the present study is retrospective and is thus vulnerable to forms of bias that are not controlled for by a retrospective design, such as selection bias; the present study also may be blind to confounding factors influencing disposition, such as social support available at home. Given these limitations, further research is needed, and a multi-center prospective study may be the best approach to further analyze SDOH which act as contributing factors to discharge disposition.

## Conclusions

Discharge disposition following one- or two-level TLIF and PLIF shows significant associations with sex and self-reported race. Male patients showed greater rates of ED visits 90 days post-surgery and are more likely to be discharged to home, whereas female patients are more likely to be discharged to SNF. Hispanic patients are also more likely to be discharged to home. No association was seen among income quartiles and home vs. non-home discharge. The trends seen between discharge disposition and both sex and race may be attributed to factors that underlie these SDOH, including but not limited to familial support, cultural differences, employment, and level of education. Additional studies are necessary to further evaluate the relationship between discharge disposition and gender, race, and income.
